# Toxoplasmosis-Associated Hemophagocytosis in a Preterm Newborn

**DOI:** 10.4274/tjh.2013.0379

**Published:** 2014-09-05

**Authors:** Sema Arayıcı, Fatma Nur Sarı, Neşe Yaralı, Mehmet Yekta Öncel, Gülsüm Kadıoğlu Şimşek, Nurdan Uras, Uğur Dilmen

**Affiliations:** 1 Zekai Tahir Burak Maternity Teaching Hospital, Division of Neonatology, Ankara, Turkey; 2 Ankara Children’s Hematology Oncology Training and Research Hospital, Clinic of Pediatric Hematology, Ankara, Turkey; 3 Yıldırım Beyazıt University Faculty of Medicine, Department of Pediatrics, Ankara, Turkey

**Keywords:** hemophagocytosis, Preterm, Toxoplasma gondii

## TO THE EDITOR

Hemophagocytic lymphohistiocytosis (HLH) is a rare, life-threatening disease characterized by inappropriate proliferation and activation of lymphocytes and macrophages in tissues, leading to multi-organ failure [1]. Primary (familial) HLH is inherited in an autosomal recessive manner. The secondary form of HLH occurs in association with infections, malignancies, rheumatologic disorders, and some metabolic diseases [2]. Although primary or secondary forms of HLH presentations during the neonatal period in term newborns have been reported previously, data on the HLH presentations of preterm newborns are scarce.

Herein, we present a 5-day-old prematurely born girl (1770 g) after 33 weeks of gestation from a primigravid mother who was antenatally diagnosed to have hydrocephalus and hepatosplenomegaly. Maternal history included no screening for intrauterine infection such as toxoplasmosis, rubella, cytomegalovirus, herpes simplex virus, and human immunodeficiency virus during pregnancy and no parental consanguinity. Physical examination revealed massive hepatosplenomegaly at birth. Laboratory findings showed hemoglobin of 14.3 g/dL, white blood cell count of 4x109/L (absolute neutrophil count 0.6x109), platelet count of 93x109/L, serum ferritin of 482 ng/mL (25-200 ng/mL), serum triglyceride of 101 mg/dL, and fibrinogen of 118 mg/dL (150-450 mg/dL). Serological screening for intrauterine infection (Toxoplasma gondii, cytomegalovirus, rubella, herpes simplex virus types 1 and 2) was negative. Cranial and abdominal ultrasonographic examination revealed hydrocephalus and hepatosplenomegaly, respectively. Since the clinical data were strongly suggestive of an intrauterine infection, further laboratory investigation was performed with polymerase chain reaction (PCR) tests. Repeated blood cultures also grew no bacteria. A bone marrow aspirate examination showed hemophagocytosis. After the diagnosis of hemaphagocytosis intravenous immune globulin was added to the conservative treatment of the newborn. On the third day of life, clinical condition of the patient deteriorated and she required mechanical ventilation and hemodynamic support. On the fifth day, the newborn died of multi-organ failure. After her death, PCR screening results were found to be Toxoplasma gondii-positive.

HLH is a reactive disorder characterized by generalized non-malignant histiocytic proliferation with prominent hemophagocytosis. There are only a few reports about preterm newborns with infection-associated hemophagocytic syndrome in the literature [3,4,5]. To the best of our knowledge, infection-associated hemophagocytic syndrome due to Toxoplasma gondii has not been previously reported in preterm newborns. Informed consent was obtained.

In our case, positive findings including splenomegaly, bicytopenia, hypofibrinogenemia, hyperferritinemia, and hemophagocytosis are considered to be associated with HLH. Although cytopenia may also be related to organomegaly associated with Toxoplasma infection, differentiation of the cause of cytopenia could not be done because of the short life span of the patient.

Although the diagnostic criteria of HLH are well defined [3], HLH in preterm newborns might not fulfill these criteria in the presence of disease since these patients usually do not have fever and, additionally, clinicians may not think of HLH diagnosis since cytopenias are a common finding of prematurity. The severe course of Toxoplasma infection in our patient, ending in mortality, may be related to the accompanying HLH. The criteria for HLH may not be fulfilled in preterm newborns and a high degree of suspicion may prevent some of the morbidity and mortalities by earlier therapeutic interventions.

## CONFLICT OF INTEREST STATEMENT

The authors of this paper have no conflicts of interest, including specific financial interests, relationships, and/ or affiliations relevant to the subject matter or materials included.
